# Rhodium-catalyzed direct alkylation of benzylic amines using alkyl bromides

**DOI:** 10.1007/s00706-018-2305-9

**Published:** 2018-11-13

**Authors:** Martin Anschuber, Robert Pollice, Michael Schnürch

**Affiliations:** 1 Institute of Applied Synthetic Chemistry, TU Wien, Getreidemarkt 9/163, 1060 Vienna, Austria; 2 Laboratorium für Organische Chemie, ETH Zürich, Vladimir-Prelog-Weg 2, 8093 Zurich, Switzerland

**Keywords:** C–H activation, C–H functionalization, Directing group, Catalysis

## Abstract

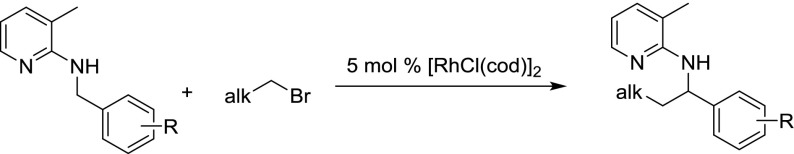

## Introduction

Direct C–H functionalization via metal catalysis is emerging as one of the most frequently investigated methods in recent years. This can be deduced from the large number of contributions published almost on a daily basis, and the number of review articles summarizing various aspects of the field [1–4]. The largest part of research is dedicated to functional group directed C–H functionalization reactions. By now literally, all of the most frequently occurring functional groups have been used as a directing group, at least in a small set of examples [5, 6]. Regarding the transformations which have been reported, the variety has increased in recent years, as well. Besides arylation, alkylation, and alkenylation reactions, also more and more C-heteroatom bond forming reactions are disclosed. Regarding alkylation reactions, olefins are the most frequently applied alkyl source [7]. However, it has to be mentioned that literature examples often limit themselves to functional group-substituted or long-chain alkenes to avoid working with gaseous reagents. We have shown, previously, that gaseous alkenes can be replaced by quaternary ammonium salts, which deliver olefins in-situ via Hofmann elimination (Scheme 1) [8].

**Figure:**
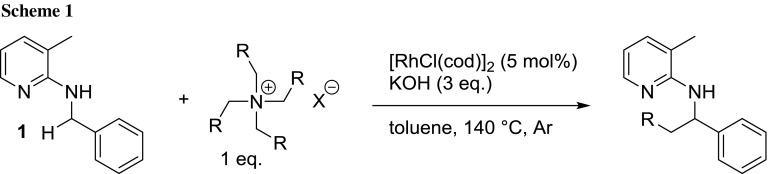

This is a very convenient protocol, since all these salts are easy-to-handle solids. Even though the quaternary ammonium salts of interest are cheap reagents, the question was raised whether alkyl halides can be used as olefin replacement, as well, since ammonium salts are typically prepared from alkyl halides and ammonia. If so, for introduction of short alkyl chains gaseous reagents could be replaced by liquid ones, which would still be more convenient than using their gaseous counterparts. We have previously investigated such a transformation briefly, and found that the reaction actually proceeds via the initial elimination to the olefin, which is then the true alkylating agent [9]. However, we then focused on mechanistic investigations of the alkylation protocol using hex-1-ene and did not investigate the substrate scope of the alkyl bromide protocol [10].

In the literature, alkyl halides are known to be suitable alkyl sources for direct alkylation reactions; however, in none of the examples, a prior elimination to an olefin is considered [11–16]. For most of them, such a mechanistic pathway can be excluded, since alkyl halide substrates which cannot give elimination to an olefin are amongst the reported examples (e.g., benzyl halides or iodomethane). However, among the reported examples, there is one contribution in which there is a high probability that initial elimination might precede the actual alkylation [17]. In this contribution, all alkyl halide examples allow the initial olefin formation, and additionally, shortcomings of the transformation could be explained, as well, since, e.g., benzylic and allylic halides failed to give the desired products (Scheme 2).

**Figure:**
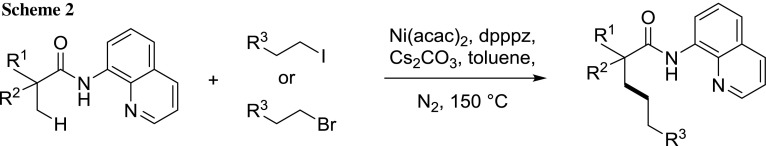

Within this contribution, we report on the development and substrate scope evaluation of an alkylation protocol using alkyl bromides initially eliminating to the corresponding terminal olefins before direct alkylation takes place (Scheme 3).

**Figure:**
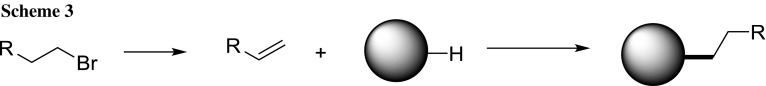

## Results and discussion

Before turning towards the substrate scope evaluation, we wanted to revisit the reaction optimization briefly. We started using the reaction conditions of our previously reported Rh(I)-catalyzed alkylation protocol [8] with **1** as substrate and 1-bromohexane instead of a quaternary ammonium salt, since these conditions were quite successful in Hofmann elimination. The first result was not very satisfactory, delivering only 12% yield of the desired product **6**, showing that the conditions for the previous protocol could not be applied when alkyl halides are the alkyl source (Table 1, entry 1). Hence, further optimization was necessary. First, we did two experiments, once increasing and once decreasing the amount of KOH. Decreasing the amount of the base to 0.5 equivalents had a detrimental effect and the yield was cut half to 6% (Table 1, entry 2). Also the threefold amount of base led only to a slightly increased yield of 16% (Table 1, entry 3). Next, the temperature was increased to 160 °C. However, also in this case, the effect was not dramatic and 22% of **6** were obtained (Table 1, entry 4). In a next step, we prolonged the reaction time, first to 46 h, then to 70 h. The resulting yields were 36% and 41%, respectively.

**Table 1:** Selected screening results for alkylation of **1** using *n*-hexyl bromide
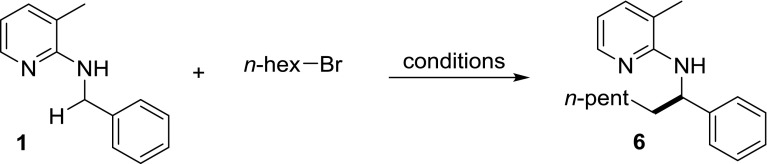

Experiment	Base	Eq. base	Catalyst/5 mol%	Temperature/°C	Time/h	Yield/%
1	KOH	1.0	[RhCl(cod)]_2_	140	22	12
2	KOH	0.5	[RhCl(cod)]_2_	140	22	6
3	KOH	3.0	[RhCl(cod)]_2_	140	22	16
4	KOH	3.0	[RhCl(cod)]_2_	160	22	22
5	KOH	3.0	[RhCl(cod)]_2_	160	46	36
6	KOH	3.0	[RhCl(cod)]_2_	160	70	41
7	K_2_CO_3_	4.5	[RhCl(cod)]_2_	160	22	50

The final step in the optimization was substituting KOH by K_2_CO_3_ and, at the same time, increasing the amount to 4.5 equivalents. The isolated yield of this reaction was 50%. Unfortunately, all other optimization experiments did not lead to a higher yield. Compared to our previously published quaternary ammonium salt protocol, this corresponds to an about 10–20% lower yield.

It has to be mentioned that this transformation is limited to the application of alkyl bromides, since alkyl chlorides did not react at all, and alkyl iodides give *N*-alkylation instead of the desired C-alkylation.

With the final conditions in hand, we started investigating the substrate scope with respect to alkyl bromides. First, we used different alkyl halides with a carbon chain length up to C22 (Scheme 4, products **2**–**14**).

**Figure:**
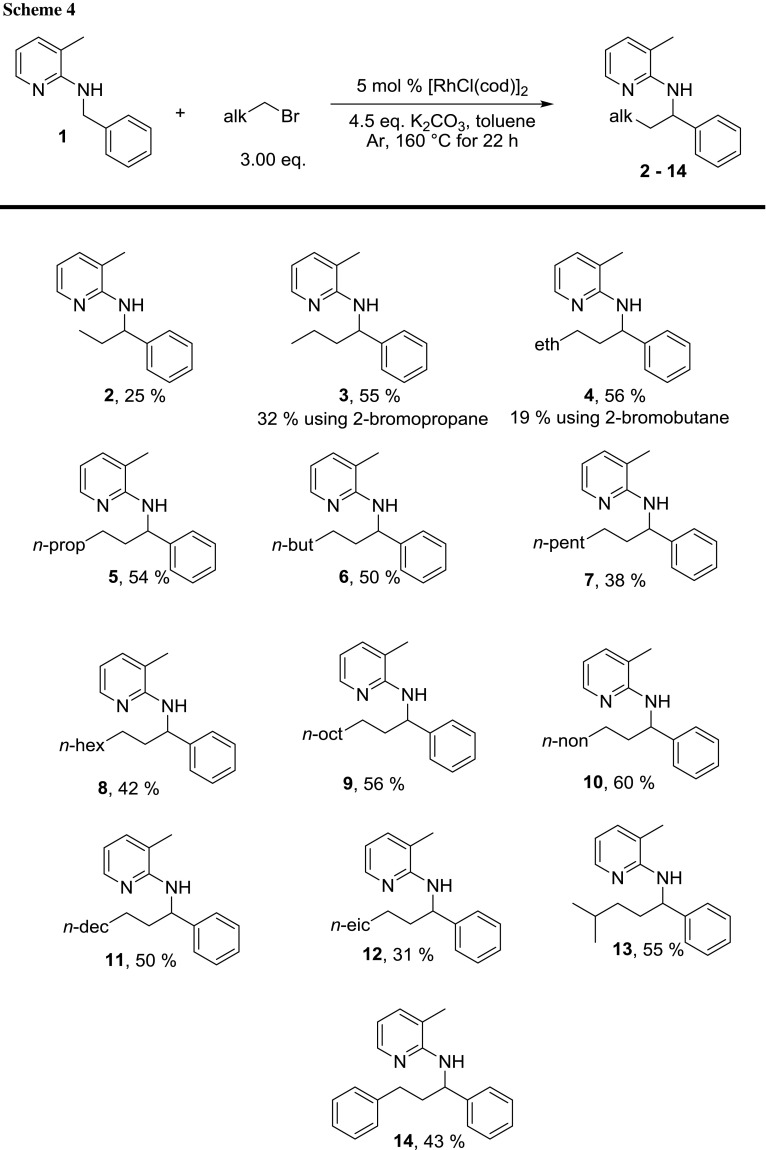

Bromoethane gave an isolated yield of only 25% of **2**. However, to synthesize **2**, N(Et_4_)Br can be used giving 68% yield [8]. Using longer alkyl bromides gave much better results, i.e., yields typically around 50%. Especially, alkyl chains from C3 to C6 and C10 to C12 gave basically the same isolated yield, i.e., they were within experimental error (Scheme 4, compounds **3**–**6**, **9**–**11**, 50–60%). Interestingly, 1-bromoheptyl and 1-bromooctyl gave a slightly lower yield (**7**, 38% and **8**, 42%), the reason for that being currently unclear. To go to the extreme, we also tested 1-bromodocosane (C22, product **12**, 31%) and observed that an alkyl bromide with such a long alkyl chain is still a potential substrate for this kind of reaction delivering the final product in 31% yield.

In addition to linear primary bromoalkanes, other alkyl bromides were tested, as well. Secondary linear alkyl bromides such as 2-bromopropane and 2-bromobutane gave the same product as their primary isomers although in considerably lower yield. This is due to the fact that the initial elimination to the corresponding olefin in case of 2-bromopropane seems to be slower, and in case of 2-bromobutane, it can additionally deliver two different olefins, of which only the terminal one can react, but the internal one is the more stable one. Using cyclohexyl bromide as substrate which can only deliver an internal olefin, no conversion was detected at all.

The reaction with 1-bromo-3-methylbutane as branched alkyl bromide delivered the product **13** in quite good yield of 55% (Scheme 4). In addition, the alkylation with 1-bromo-2-phenylethane, which delivers styrene upon elimination, worked with an acceptable yield of **14** (Scheme 3, 43%). These two substrates are not accessible for this reaction via quaternary ammonium salts.

Next, it was tested whether the developed alkylation protocol is limited to unsubstituted alkyl halides, since utility and acceptance of the protocol would depend largely on the possibility of general applicability. It was hypothesized that other alkylation reactions using olefins should be accessible to our protocol as long as the moieties of the substrates do not deactivate or destroy the catalyst. However, it turned out that alkyl bromides eliminating to substituted allylic and vinylic compounds were not tolerated giving either no or very low conversion (Scheme 5).

**Figure:**
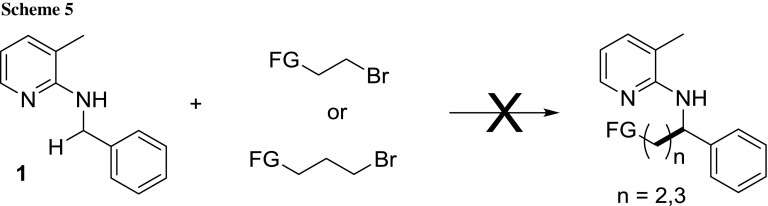

In cases where the formed olefin is further away from the functional group, the reaction worked again as demonstrated by the reaction of ethyl 4-bromobutanoate and ethyl 5-bromopentanoate, which delivered the respective products in 57% and 35% yield, respectively (Scheme 6, compounds **18** and **19**).

**Figure:**
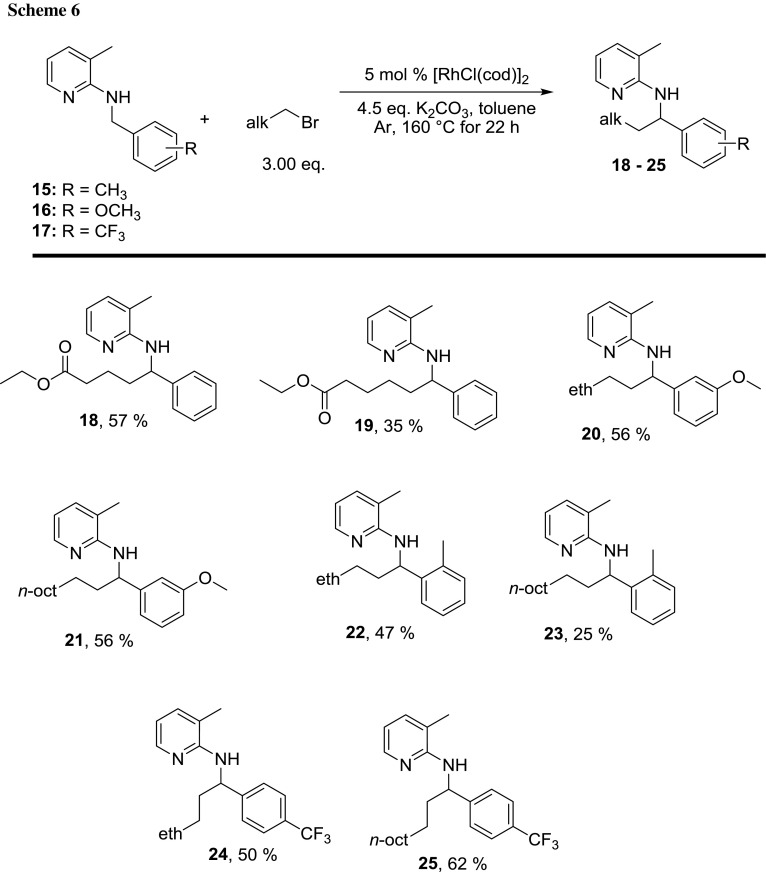

Furthermore, we changed the substitution pattern at the *ortho*-, *meta*-, and *para*-position of the benzyl group of our starting material. Substrate **15** is bearing an *o*-methyl group, **16** is bearing an *m*-methoxy group, and **17** is bearing a *p*-trifluoromethyl group. The rationale behind using these groups is to find out whether the reaction is influenced by sterically demanding groups, as well as both electron donating and withdrawing groups.

These substrates were tested in *n*-butylation and *n*-decylation reactions, respectively, to demonstrate the utility of our protocol to introduce both a short and a long alkyl chain. As can be seen in Scheme 6 (compounds **20**–**25**), again, the same range of yields was obtained (47–62%), apart from one reaction (vide infra). It was observed that the methyl group at the benzylic *ortho*-position decreased the yield compared to the unsubstituted starting compound **1** (Scheme 6, compounds **22** and **23**). This can be rationalized using steric arguments. The decrease in yield is much more dramatic in case of 1-bromodecane as coupling partner as compared to 1-bromobutane, which again hints towards a steric effect.

A *m*-methoxy group gave good yields of 56% in both cases (Scheme 6, compounds **20** and **21**). In addition, an electron-withdrawing substituent such as a *p*-CF_3_ group seems to have no impact on the isolated yield, since 50% of **24** and 62% of **25** were obtained, respectively.

Finally, it has to be mentioned that the cleavage of the directing group is possible following a strategy that has proven to be successful in the literature [18] and in our lab [19–21].

## Conclusion

We demonstrated that alkyl bromides can be used as olefin precursors in the direct alkylation of C(sp^3^)–H bonds of benzylic amines. The reaction works with slightly lower efficiency as the previously disclosed protocol using quaternary ammonium salts [8]. Linear and unsubstituted alkyl bromides give the best results and examples up to a C22 chain length were reported. Functional group tolerance on the amine substrate was good, but limitations were observed when substituted alkyl bromides were used. If elimination to an allylic or vinylic olefin would need to occur, the reaction is inefficient. In addition, not many functional groups were tolerated. However, carboxylic acid esters work well. This gives a handle for further elaboration of the obtained products. Finally, cleavage of the directing group is possible, as well, an important feature in DG-assisted C–H functionalization.

## Experimental

In general, unless noted otherwise, chemicals were purchased from commercial suppliers and used without further purification. Cyclooctadiene rhodium chloride dimer [RhCl(cod)]_2_ was handled in the glovebox under argon. Dry and degassed toluene was stored over molecular sieves in the glovebox under argon. Other dry solvents were obtained by passing pre-dried material through a cartridge-containing activated alumina (solvent dispensing system) and stored under nitrogen atmosphere until usage.

**Fig. 1:**
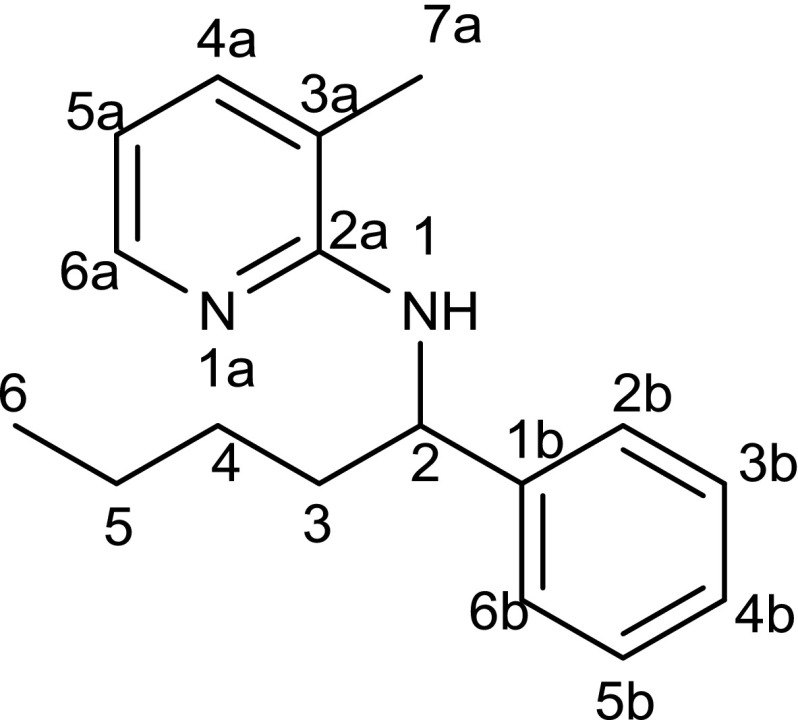
Scheme for assigning NMR signals

^1^H NMR, ^13^C NMR, and HSQC spectra were recorded on a Bruker Avance 400; chemical shifts are reported in ppm, using Me_4_Si as internal standard. NMR signals were assigned according to Fig. 1. GC–MS was performed on a Thermo Trace 1300 GC/MS ISQ LT (quadrupole, EI+) with a TR-5 capillary column (7 m × 0.32 mm, 0.25 μm film, achiral). Temperature program: Start at 100 °C (hold 2 min), 35 °C/min, and 300 °C (hold 4 min). GC spectra were recorded on a Thermo Focus GC using a BGB-5 capillary column (30 m × 0.32 mm, 1.0 μm film, achiral) with the following oven temperature program: Start at 100 °C (hold 2 min), 35 °C/min, 300 °C (hold 4 min). For TLC aluminum, backed silica gel 60 with fluorescence indicator F254 was used. Column chromatography was performed on Silica 60 from Merck (40–63 μm). Flash chromatography was carried out on a Büchi Sepacore™ MPLC system. Melting points were determined on an automated melting point system (Büchi Melting Point B-545). High-resolution mass spectrometry (HRMS) for the literature-unknown compounds was performed by liquid chromatography in combination with hybrid ion trap and high-resolution time-of-flight–mass spectrometry (LC–IT–TOF–MS) in only positive-ion detection mode with the recording of standard (MS) and tandem (MS/MS) spectra.

### General procedure A for C–H activation reactions to compounds **2**–**14** and **18**–**25**

Solid starting materials (except the catalyst) were placed in an oven-dried 8 cm^3^ glass vial with a solid top screw cap and a magnetic stirring bar. The vial was transferred into the glovebox under argon. Catalyst, liquid starting materials, solvent, and dodecane (as internal standard) were added in the glovebox. Finally, the vial was closed and the reaction mixture was heated in a heating block for the desired time at the desired temperature.

### General work-up procedure B for C–H activation reactions towards compounds **2**–**14** and **18**–**25**

After cooling the reaction mixture to room temperature, the solid material was removed by filtration using a Pasteur pipette with cotton and Hyflo. The residue was washed with CH_2_Cl_2_. The combined organic filtrate was concentrated under reduced pressure. The resulting crude residue was purified by flash column chromatography (LP/EtOAc).

### General procedure C for precursor synthesis (1, 15–17)

Solid starting materials were placed in a 100 cm^3^ 3-necked-flask, evacuated, and flushed with argon three times. Then, the liquid starting materials and, finally, toluene were added through the septum with a syringe. The mixture was heated at 130 °C (oil bath temperature) maintaining the argon atmosphere with a balloon. The reaction was stopped after 18 h (TLC). After cooling to r.t., the solid material was removed by filtration and washed with CH_2_Cl_2_. The combined organic layers were evaporated and the resulting crude product was purified by flash column chromatography (LP/EtOAc) starting with 5% EtOAc to 10% EtOAc over the course of 20 min. Then, flash column chromatography was continued with 10% EtOAc. Drying under reduced pressure delivered the pure product.

#### *N*-Benzyl-3-methylpyridin-2-amine (**1**)

In a 100 cm^3^ three-necked-flask, were placed 67 mg Pd(OAc)_2_ (0.3 mmol, 0.02 eq.), 187 mg rac. BINAP (0.3 mmol, 0.02 eq.), and 7.214 g K_2_CO_3_ (52.5 mmol, 3.5 eq.), and the flask was evacuated and flushed with argon three times. Then, 1.63 cm^3^ 2-chloro-3-methylpyridine (15 mmol, 1 eq.), 1.97 cm^3^ freshly distilled benzylamine (18 mmol, 1.2 eq.), and, finally, 38 cm^3^ toluene were added through the septum with a syringe. The mixture was heated to 130 °C maintaining the argon atmosphere with a balloon. The reaction was stopped after 18 h (TLC). After cooling to r.t. the solid material was removed by filtration and washed with 150 cm^3^ CH_2_Cl_2_. The combined organic layers were evaporated and the resulting crude product was purified by flash column chromatography (LP/EtOAc) starting with 5% EtOAc to 10% EtOAc over the course of 20 min. Then, flash column chromatography was continued with 10% EtOAc. Drying under reduced pressure delivered 2.55 g (86%) **1** as beige solid. Analytical data are in accordance to the literature [22].

#### 3-Methyl-*N*-(1-phenylpropyl)pyridin-2-amine (**2**)

The reaction was carried out according to general procedure A with 100 mg **1** (0.50 mmol, 1 eq.), 163 mg 1-bromoethane (1.50 mmol, 3 eq.), 311 mg K_2_CO_3_ (2.25 mmol, 4.5 eq.), and 12 mg [RhCl(cod)]_2_ (0.025 mmol, 0.05 eq.) in 2 cm^3^ dry and degassed toluene. The reaction mixture was heated for 22 h at 160 °C. The general work-up procedure B for C–H activation reactions was followed. The combined organic layers were evaporated and the resulting crude product was purified by flash column chromatography (LP/EtOAc, 45 g SiO_2_, flowrate 30 cm^3^/min) starting with pure LP for 10 min and then continuing using a gradient which varies the solvents from 0% to 5% EtOAc within 45 min. The product was dried under reduced pressure and **2** was isolated in 25% (29 mg) yield as yellow oil. Analytical data are in accordance to the literature [19].

#### 3-Methyl-*N*-(1-phenylbutyl)pyridin-2-amine (**3**)

The reaction was carried out according to general procedure A with 100 mg **1** (0.50 mmol, 1 eq.), 184 mg 2-bromopropane (1.50 mmol, 3 eq.), 311 mg K_2_CO_3_ (2.25 mmol, 4.5 eq.), and 12 mg [RhCl(cod)]_2_ (0.025 mmol, 0.05 eq.) in 2 cm^3^ dry and degassed toluene. The reaction mixture was heated for 22 h at 160 °C. The general work-up procedure B for C–H activation reactions was followed. The combined organic layers were evaporated and the resulting crude product was purified by flash column chromatography (LP/EtOAc, 45 g SiO_2_, flowrate 30 cm^3^/min) using a gradient which varies the solvents from 0% to 5% EtOAc within 45 min. Drying under reduced pressure delivered **3** in 32% (39 mg) yield. TLC: *R*_f_ = 0.74 (LP/EtOAc 10:1); analytical data are in accordance to the literature [8].

#### 3-Methyl-*N*-(1-phenylpentyl)pyridin-2-amine (**4**)

The reaction was carried out according to general procedure A with 100 mg **1** (0.50 mmol, 1 eq.), 206 mg 1-bromobutane (1.50 mmol, 3 eq.), 311 mg K_2_CO_3_ (2.25 mmol, 4.5 eq.), and 12 mg [RhCl(cod)]_2_ (0.025 mmol, 0.05 eq.) in 2 cm^3^ dry and degassed toluene. The reaction mixture was heated for 22 h at 160 °C. The general work-up procedure B for C–H activation reactions was followed. The combined organic layers were evaporated and the resulting crude product was purified by flash column chromatography (LP/EtOAc, 45 g SiO_2_, flowrate 30 cm^3^/min) using a gradient which varies the solvents from 0% to 5% EtOAc within 45 min. The product was dried under reduced pressure and **4** was isolated in 56% (71 mg) yield as pale yellowish oil. Analytical data are in accordance to the literature [8].

#### 3-Methyl-*N*-(1-phenylhexyl)pyridin-2-amine (**5**)

The reaction was carried out according to general procedure A with 100 mg **1** (0.50 mmol, 1 eq.), 225 mg 1-bromopentane (1.50 mmol, 3 eq.), 311 mg K_2_CO_3_ (2.25 mmol, 4.5 eq.), and 12 mg [RhCl(cod)]_2_ (0.025 mmol, 0.05 eq.) in 2 cm^3^ dry and degassed toluene. The reaction mixture was heated for 22 h at 160 °C. The general work-up procedure B for C–H activation reactions was followed. The combined organic layers were evaporated and the resulting crude product was purified by flash column chromatography (LP/EtOAc, 45 g SiO_2_, flowrate 30 cm^3^/min) starting with pure LP for 10 min. Then, the flash column chromatography was continued using a gradient which varies the solvents from 0% to 10% EtOAc within 45 min. The product was dried under reduced pressure and **5** was isolated in 35% (46 mg) yield as pale yellowish oil. Analytical data are in accordance to the literature [8].

#### 3-Methyl-*N*-(1-phenylheptyl)pyridin-2-amine (**6**)

The reaction was carried out according to general procedure A with 100 mg **1** (0.50 mmol, 1 eq.), 248 mg 1-bromohexane (1.50 mmol, 3 eq.), 311 mg K_2_CO_3_ (2.25 mmol, 4.5 eq.), and 12 mg [RhCl(cod)]_2_ (0.025 mmol, 0.05 eq.) in 2 cm^3^ dry and degassed toluene. The reaction mixture was heated for 22 h at 160 °C. The general work-up procedure B for C–H activation reactions was followed. The combined organic layers were evaporated and the resulting crude product was purified by flash column chromatography (LP/EtOAc, 45 g SiO_2_, flowrate 30 cm^3^/min) starting with pure LP for 10 min. Then, the flash column chromatography was continued using a gradient which varies the solvents from 0% to 10% EtOAc within 45 min. The product was dried under reduced pressure and **6** was isolated in 40% (56 mg) yield as pale yellowish oil. Analytical data are in accordance to the literature [8].

#### 3-Methyl-*N*-(1-phenyloctyl)pyridin-2-amine (7, C_20_H_28_N_2_)

The reaction was carried out according to general procedure A with 100 mg **1** (0.50 mmol, 1 eq.), 269 mg 1-bromoheptane (1.50 mmol, 3 eq.), 311 mg K_2_CO_3_ (2.25 mmol, 4.5 eq.), and 12 mg [RhCl(cod)]_2_ (0.025 mmol, 0.05 eq.) in 2 cm^3^ dry and degassed toluene. The reaction mixture was heated for 22 h at 160 °C. The general work-up procedure B for C–H activation reactions was followed. The combined organic layers were evaporated and the resulting crude product was purified by flash column chromatography (LP/EtOAc, 45 g SiO_2_, flowrate 30 cm^3^/min) starting with pure LP for 5 min. Then, the flash column chromatography was continued using a gradient which varies the solvents from 0% to 5% EtOAc within 1 h. Drying under reduced pressure delivered **7** in 38% (57 mg) yield as yellow oil. TLC: *R*_f_ = 0.59 (LP/EtOAc 10:1); ^1^H NMR (400 MHz, CDCl_3_): *δ* = 0.86 (t, *J* = 14.6, 9.8 Hz, 3H, C[9]–H_3_), 1.14–1.38 (m, 10H, C[4–8]–H_2_), 1.87 (ddd, *J* = 22.3, 9.6, 6.8 Hz, 1H, C[3]–H), 2.12 (s, 3H, C[7a]–H_3_), 4.37 (d, *J* = 7.6 Hz, 1H, N–H), 5.24 (d, *J* = 7.3 Hz, 1H, C[2]–H), 6.47 (dd, *J* = 7.1, 5.1 Hz, 1H, C[5a]–H), 7.15–7.25 (m, 2H, C[4a; 4b]–H), 7.27–7.41 (m, 4H, C[2b; 3b; 5b; 6b]–H), 7.95 (dd, *J* = 5.0, 1.8 Hz, 1H, C[6a]–H) ppm; ^13^C NMR (101 MHz, CDCl_3_): *δ* = 14.1 (q, C[9]), 17.1 (q, C[7a]), 22.7 (t, C[8]), 26.4 (t, C[7]), 29.2 (t, C[6]), 29.6 (t, C[5]), 31.8 (t, C[4]), 37.6 (t, C[3]), 54.6 (d, C[2]), 112.5 (d, C[5a]), 116.2 (s, C[3a]), 126.5 (d, C[2b; 6b]), 126.7 (d, C[4b]), 128.4 (d, C[3b; 5b]), 136.7 (d, C[4a]), 144.6 (s, C[1b]), 145.6, (d, C[6a]), 156.2 (s, C[2a]) ppm; GC–MS: retention time: 7.86 min; main fragments: *m/z* (%) = 296 (M^+^, 11), 211 (19), 197 (100), 108 (22), 91 (21), 65 (11).

#### 3-Methyl-*N*-(1-phenylnonyl)pyridin-2-amine (**8**)

The reaction was carried out according to general procedure A with 100 mg **1** (0.50 mmol, 1 eq.), 290 mg 1-bromooctane (1.50 mmol, 3 eq.), 311 mg K_2_CO_3_ (2.25 mmol, 4.5 eq.), and 12 mg [RhCl(cod)]_2_ (0.025 mmol, 0.05 eq.) in 2 cm^3^ dry and degassed toluene. The reaction mixture was heated for 22 h at 160 °C. The general work-up procedure B for C–H activation reactions was followed starting with pure LP for 10 min. Then, the flash column chromatography was continued using a gradient which varies the solvents from 0% to 5% EtOAc within 1 h. Drying under reduced pressure delivered **8** in 42% (65 mg) yield as yellow oil. TLC: *R*_f_ = 0.51 (LP/EtOAc 10:1); analytical data are in accordance to the literature [8].

#### 3-Methyl-*N*-(1-phenylundecyl)pyridin-2-amine (9, C_23_H_34_N_2_)

The reaction was carried out according to general procedure A with 100 mg **1** (0.50 mmol, 1 eq.), 332 mg 1-bromodecane (1.50 mmol, 3 eq.), 311 mg K_2_CO_3_ (2.25 mmol, 4.5 eq.), and 12 mg [RhCl(cod)]_2_ (0.025 mmol, 0.05 eq.) in 2 cm^3^ dry and degassed toluene. The reaction mixture was heated for 22 h at 160 °C. The general work-up procedure B for C–H activation reactions was followed using a gradient which varies the solvents from 0% to 5% EtOAc within 1 h. Drying under reduced pressure delivered **9** in 56% (65 mg) yield. TLC: *R*_f_ = 0.57 (LP/EtOAc 10:1); ^1^H NMR (400 MHz, CDCl_3_): *δ* = 0.88 (t, *J* = 6.8 Hz, 3H, C[12]–H_3_), 1.10–1.48 (m, 16H, C[4–11]–H_2_), 1.77–1.98 (m, 2H, C[3]–H_2_), 2.12 (s, 3H, C[7a]–H_3_), 4.37 (d, *J* = 7.4 Hz, 1H, N–H), 5.24 (q, *J* = 7.1 Hz, 1H, C[2]–H), 6.47 (dd, *J* = 7.1, 5.0 Hz, 1H, C[5a]–H), 7.15–7.27 (m, 2H, C[4a; 4b]–H), 7.27–7.44 (m, 4H, C[2b; 3b; 5b; 6b]–H), 7.96 (dd, *J* = 5.1, 1.8 Hz, 1H, C[6a]–H) ppm; ^13^C NMR (101 MHz, CDCl_3_): *δ* = 14.1 (q, C[12]), 17.1 (q, C[7a]), 22.7 (t, C[6]), 26.4 (t, C[7]), 29.3 (t, C[5]), 29.5 (t, C[8, 9]), 29.6 (t, C[4a; 4b]), 31.9 (t, C[11]), 37.6 (t, C[3]), 54.6 (d, C[2]), 112.5 (d, C[5a]), 116.1 (s, C[3a]), 126.5 (d, C[2b; 6b]), 126.7 (d, C[4b]), 128.1 (d, C[3b; 5b]), 136.8 (d, C[4a]), 144.6 (s, C[1b]), 145.8 (d, C[6a]), 156.2 (s, C[2a]) ppm; GC–MS: retention time: 8.73 min; main fragments: *m/z* (%) = 338 (M^+^, 8), 211 (20), 197 (100), 108 (23), 91 (18), 65 (7).

#### 3-Methyl-*N*-(1-phenyldodecyl)pyridin-2-amine (10, C_24_H_36_N_2_)

The reaction was carried out according to general procedure A with 100 mg **1** (0.50 mmol, 1 eq.), 353 mg 1-bromoundecane (1.50 mmol, 3 eq.), 311 mg K_2_CO_3_ (2.25 mmol, 4.5 eq.), and 12 mg [RhCl(cod)]_2_ (0.025 mmol, 0.05 eq.) in 2 cm^3^ dry and degassed toluene. The reaction mixture was heated for 22 h at 160 °C. The general work-up procedure B for C–H activation reactions was followed starting with pure LP for 10 min. Then, the flash column chromatography was continued using a gradient which varies the solvents from 0% to 5% EtOAc within 1 h. Drying under reduced pressure delivered **10** in 60% (106 mg) yield. TLC: *R*_f_ = 0.55 (LP/EtOAc 10:1); ^1^H NMR (400 MHz, CDCl_3_): *δ* = 0.93 (s, 3H, C[13]–H_3_), 1.28 (s, 18H, C[4–12]–H_2_), 1.81–2.03 (m, 2H, C[3]–H_2_), 2.15 (s, 3H, C[7a]–H_3_), 4.43 (s, 1H, N–H), 5.30 (q, *J* = 7.1 Hz, 1H, C[2]–H), 6.50 (dd, *J* = 7.1, 5.1 Hz, 1H, C[5a]–H), 7.19–7.30 (m, 2H, C[4a; 4b]–H)), 7.30–7.45 (m, 4H, C[2b; 3b; 5b; 6b]–H), 8.00 (dd, *J* = 5.0, 1.9 Hz, 1H, C[6a]–H) ppm; ^13^C NMR (101 MHz, CDCl_3_): *δ* = 14.2 (q, C[13]), 17.1 (q, C[7a]), 29.6 (t, C[5–10]), 32.0 (t, C[11]), 37. 6 (t, C[3]), 54.7 (d, C[2]), 112.5 (d, C[5a]), 116.2 (s, C[3a]), 126.5 (d, C[2b; 6b]), 126.7 (d, C[4b]), 128. 4 (d, C[3b; 5b]), 136.8 (d, C[4a]), 144.6 (s, C[1b]), 145.5 (d, C[6a]), 156.2 (s, C[2a]) ppm; GC–MS: retention time: 8.73 min; main fragments: *m/z* (%) = 352 (M^+^, 8), 211 (19), 197 (100), 108 (23), 91 (19), 65 (7); HRMS: *m/z* calculated for C_24_H_36_N_2_ ([M+H]^+^) 353.2957, found 353.2972; Δ = 5.93 ppm.

#### 3-Methyl-*N*-(1-phenyltridecyl)pyridin-2-amine (11, C_25_H_38_N_2_)

The reaction was carried out according to general procedure A with 100 mg **1** (0.50 mmol, 1 eq.), 374 mg 1-bromododecane (1.50 mmol, 3 eq.), 311 mg K_2_CO_3_ (2.25 mmol, 4.5 eq.), and 12 mg [RhCl(cod)]_2_ (0.025 mmol, 0.05 eq.) in 2 cm^3^ dry and degassed toluene. The reaction mixture was heated for 22 h at 160 °C. The general work-up procedure B for C–H activation reactions was followed using first 5 min pure LP and continuing using a gradient which varies the solvents from 0% to 5% EtOAc within 1 h. Drying delivered **24** in 56% (96 mg) yield as pale yellowish oil. TLC: *R*_f_ = 0.9 (LP/EtOAc 10:1); ^1^H NMR (400 MHz, CDCl_3_): *δ* = 0.88 (t, *J* = 6.8 Hz, 3H, C[14]–H_3_), 1.21–1.36 (m, 20H, C[4–13]–H_2_), 1.78–1.97 (m, 2H, C[3]–H_2_), 2.12 (s, 3H, C[7a]–H_3_), 4.41 (s, 1H, N–H), 5.26 (q, *J* = 7.3 Hz, 1H, C[2]–H), 6.47 (dd, *J* = 7.1, 5.1 Hz, 1H, C–[5a]–H), 7.18–7.25 (m, 2H, C[4a; 4b]–H), 7.31 (t, *J* = 7.5 Hz, 2H, C[2b; 6b]–H, 7.37 (d, *J* = 7.5 Hz, 2H, C[3b; 5b]–H), 7.95 (dd, *J* = 5.1, 1.7 Hz, 1H, C[6a]–H) ppm; ^13^C NMR (101 MHz, CDCl_3_): *δ* = 14.14 (q, C[14]), 17.09 (q, C[7a]), 22.71 (t, C[13]), 26.35 (t, C[12]), 29.31–29.75 (m) (t, C[5–11]), 31.94 (t, C[4]), 37.55 (t, C[3]), 54.72 (d, C[2]), 112.50 (d C[5a]), 126.53 (d, C[3b; 5b]), 126.73 (d, C[4b]), 128.39 (d, C[2b; 6b]), 136.91 (d, C[4a]), 144.43 (s, C[1b]) ppm; GC–MS: retention time: 9.22 min; main fragments: *m/z* (%) = 366 (M^+^, 8), 267 (1), 211 (19), 197 (100), 108 (26), 91 (18), 65 (6); HRMS: *m/z* calculated for C_25_H_38_N_2_ ([M+H]^+^) 367.3114, found 367.3119; ∆ = 3.14 ppm.

#### 3-Methyl-*N*-(1-phenyltricosyl)pyridin-2-amine (12, C_35_H_58_N_2_)

The reaction was carried out according to general procedure A with 100 mg **1** (0.50 mmol, 1 eq.), 584 mg 1-bromodocosane (1.50 mmol, 3 eq.), 311 mg K_2_CO_3_ (2.25 mmol, 4.5 eq.), and 12 mg [RhCl(cod)]_2_ (0.025 mmol, 0.05 eq.) in 2 cm^3^ dry and degassed toluene. The reaction mixture was heated for 22 h at 160 °C. The general work-up procedure B for C–H activation reactions was followed using a gradient which varies the solvents from 0% to 20% EtOAc within 45 min. Upon recrystallisation, the pure product was isolated in 31% (80 mg) yield as white crystals. M.p.: 47.5–47.6 °C; TLC: *R*_f_ = 0.8 (LP/EtOAc 5:1); ^1^H NMR (400 MHz, CDCl_3_): *δ* = 0.88 (t, *J* = 6.7 Hz, 3H, C[24]–H_3_),), 1.19–1.33 (m, 40H, C[4–23]–H_2_), 1.80–1.90 (m, 2H, C[3]–H_2_), 2.13 (s, 3H, C[7a]–H_3_), 4.52 (s, 1H, N–H), 5.32 (s, 1H, C[2]–H), 6.49 (dd, *J* = 7.1, 5.2 Hz, 1H, C[5a]–H), 7.18–7.25 (m, 2H, C[4a; 4b]–H), 7.31 (t, *J* = 7.5 Hz, 2H, C[2b; 6b]–H), 7.39 (d, *J* = 7.5 Hz, 2H, C[3b; 5b]–H), 7.95 (dd, *J* = 5.2, 1.7 Hz, 1H, C[6a]–H) ppm; ^13^C NMR (101 MHz, CDCl_3_): *δ* = 14.1 (q, C[24]), 17.1 (q, C[7a]), 22.7 (t, C[23]), 26.3 (t, C[4]), 29.81–29.32 (m) (t, C[5–21]), 31.9 (t, C[22]), 37.6 (t, C[3]), 55.0 (d, C[2]), 112.5 (d, C[5a]), 126.5 (d, C[3b; 5b]), 126.9 (d, C[4b]), 128.5 (d, C[2b; 6b]) ppm; LC–MS: retention time: 2.28 min; main fragments: *m/z* (%) = 507.40 ([M+H]^+^, 100), 508.4 (40), 509.4 (7), 493.4 (18), 322.2 (16), 314.2 (10), 282.2 (28).

#### 3-Methyl-*N*-(4-methyl-1-phenylpentyl)pyridin-2-amine (13, C_18_H_24_N_2_)

The reaction was carried out according to general procedure A with 100 mg **1** (0.50 mmol, 1 eq.), 227 mg 1-bromo-3-methylbutane (1.50 mmol, 3 eq.), 311 mg K_2_CO_3_ (2.25 mmol, 4.5 eq.), and 12 mg [RhCl(cod)]_2_ (0.025 mmol, 0.05 eq.) in 2 cm^3^ dry and degassed toluene. The reaction mixture was heated for 22 h at 160 °C. The general work-up procedure B for C–H activation reactions was followed. The combined organic layers were evaporated and the resulting crude product was purified by flash column chromatography (LP/EtOAc, 45 g SiO_2_, flowrate 30 cm^3^/min) starting with pure LP for 10 min. Then, the flash column chromatography was continued using a gradient which varies the solvents from 0% to 5% EtOAc within 45 min. Drying under reduced pressure delivered **13** in 55% (90 mg) yield as pale yellowish oil. TLC: *R*_f_ = 0.67 (LP/EtOAc 10:1); ^1^H NMR (400 MHz, CDCl_3_): *δ* = 0.87 (dd, *J* = 6.6, 3.5 Hz, 6H, C[6, 7]–H_3_), 1.09–1.39 (m, 2H, C[3]–H_2_), 1.58 (dp, *J* = 13.3, 6.7 Hz, 1H, C[5]–H), 1.73–2.00 (m, 2H, C[4]–H_2_), 2.12 (s, 3H, C[7a]–H_3_), 4.39 (d, *J* = 7.5 Hz, 1H, N–H), 5.23 (q, *J* = 7.2 Hz, 1H, C[2]–H), 6.47 (dd, *J* = 7.1, 5.1 Hz, 1H, C[5a]–H), 7.14–7.25 (m, 2H, C[4a; 4b]–H), 7.27–7.42 (m, 4H, C[2b; 3b; 5b; 6b]–H), 7.95 (dd, *J* = 6.0, 1.8 Hz, 1H, C[6a]–H) ppm; ^13^C NMR (101 MHz, CDCl_3_): *δ* = 17.2 (q, C[6, 7]), 22.7 (q, C[7a]), 28.2 (d, C[5]), 35.4 (t, C[4]), 35.6 (t, C[3]), 55.0 (d, C[2]), 112.6 (d, C[5a]), 116.4 (s, C[3a]), 126.7 (d, C[2b; 6b]), 126.9 (d, C[4b]), 128.5 (d, C[3b; 5b]), 137.0 (d, C[4a]), 144.6 (s, C[1b]), 145.5 (d, C[6a]), 156.2 (s, C[2a]) ppm; GC–MS: retention time: 9.97 min; main fragments: *m/z* (%) = 268 (M^+^, 6), 211 (17), 197 (100), 108 (30), 91 (20).

#### *N*-(1,3-Diphenylpropyl)-3-methylpyridin-2-amine (14, C_21_H_22_N_2_)

The reaction was carried out according to general procedure A with 100 mg **1** (0.50 mmol, 1 eq.), 278 mg (2-bromoethyl)benzene (1.50 mmol, 3 eq.), 311 mg K_2_CO_3_ (2.25 mmol, 4.5 eq.), and 12 mg [RhCl(cod)]_2_ (0.025 mmol, 0.05 eq.) in 2 cm^3^ dry and degassed toluene. The reaction mixture was heated for 22 h at 160 °C. The general work-up procedure B for C–H activation reactions was followed. The combined organic layers were evaporated and the resulting crude product was purified by flash column chromatography (LP/EtOAc, 45 g SiO_2_, flowrate 30 cm^3^/min) starting with pure LP for 10 min. Then, the flash column chromatography was continued using a gradient which varies the solvents from 0% to 5% EtOAc within 45 min. Drying under reduced pressure delivered **14** in 23% (36 mg) yield as pale yellowish oil. TLC: *R*_f_ = 0.46 (LP/EtOAc 10:1); ^1^H NMR (400 MHz, CDCl_3_): *δ* = 1.95 (s, 3H, C[7a]–H_3_), 1.96–2.28 (m, 2H, C[4]–H), 2.70 (dddd, *J* = 51.9, 14.0, 9.8, 6.0 Hz, 2H, C[3]–H_2_), 4.27–4.34 (m, 1H, N–H), 5.30 (q, *J* = 7.1 Hz, 1H, C[2]–H), 6.40 (dd, *J* = 7.1, 5.1 Hz, 1H, C[5a]–H), 7.04–7.28 (m, 9H, C[2b; 3b; 5b; 6b; 2c–6c]–H), 7.28–7.36 (m, 2H, C[4a; 4b]–H), 7.88 (dd, *J* = 5.2, 1.8 Hz, 1H, C[6a]–H) ppm; ^13^C NMR (101 MHz, CDCl_3_): *δ* = 17.0 (q, C[7]), 32.8 (t, C[4]), 38.9 (t, C[3]), 54.7 (d, C[2]), 112.7 (s, C[3a]), 116.4 (d, C[5a]), 125.9 (d, C[2b; 6b]), 126.6 (d, C[4b]), 127.0 (d, C[3b; 5b]), 127.7 (d, C[4c]), 128.5 (d, C[3c; 5c]), 128.6 (d, C[2c; 6c]), 136.9 (d, C[4a]), 142.1 (s, C[1c]), 144.1 (s, C[1b]), 145.4 (d, C[6a]), 156.0 (s, C[2a]) ppm; GC–MS: retention time: 8.50 min; main fragments: *m/z* (%) = 302 (M^+^, 9), 211 (85), 197 (100), 108 (20), 91 (52), 65 (30).

#### 3-Methyl-*N*-(2-methylbenzyl)pyridin-2-amine (15, C_14_H_16_N_2_)

The reaction was carried out according to general procedure C with 67 mg Pd(OAc)_2_ (0.3 mmol, 0.02 equiv.), 187 mg rac. BINAP (0.3 mmol, 0.02 equiv.), 7.26 g K_2_CO_3_ (52.5 mmol, 3.5 equiv.), 1.91 g 2-chloro-3-methylpyridine (15 mmol, 1 equiv.), 2.18 g 2-methylbenzylamine (18 mmol, 1.2 equiv.), and 38 cm^3^ toluene. The yield of the thereby prepared yellow crystals is 73% (2.34 g). TLC: *R*_f_ = 0.2 (LP/EtOAc 10:1); ^1^H NMR (400 MHz, CDCl_3_): *δ* = 2.07 (s, 3H, C[7a]–H3), 2.39 (s, 3H, C[7b]–H3), 4.20 (s, 1H, N[1]–H), 4.66 (d, *J* = 4.9 Hz, 2H, C[2]–H_2_), 6.57 (dd, *J* = 7.0, 5.2 Hz, 1H, C[5a]–H), 7.20 (m, 3H, C[4a; 2b; 4b]), 7.25 (d, *J* = 6.6 Hz, 1H, C[3b]), 7.34 (d, *J* = 6.6 Hz, 1H, C[5b]), 8.07 (d, *J* = 4.2 Hz, 1H, C[6a]) ppm; ^13^C NMR (101 MHz, CDCl_3_): *δ* = 17.1 (q, C[7a]), 19.2 (q, C[7b]), 44.3 (t, C[2]), 112.9 (d, C[5a]), 116.7 (s, C[3a]), 126.2 (d, C[2b]), 127.6 (d, C[4b]), 128.8 (d, C[5b]), 130.6 (d, C[4a]), 136.9 (s, C[2b]), 137.0 (d, C[3b]), 137.6 (s, C[1b]) 145.4 (d, C[6a]), 156.7 (s, C[2a]) ppm.

#### *N*-(3-Methoxybenzyl)-3-methylpyridin-2-amine (16, C_14_H_16_N_2_O)

The reaction was carried out according to general procedure C with 29 mg Pd(OAc)_2_ (0.13 mmol, 0.02 equiv.), 77 mg rac. BINAP (0.12 mmol, 0.02 equiv.), 2.93 g K_2_CO_3_ (21.2 mmol, 3.5 equiv.), 0.78 g 2-chloro-3-methylpyridine (6.1 mmol, 1 equiv.), 1.0 g 3-methoxybenzylamine (7.3 mmol, 1.2 equiv.), and 30 cm^3^ toluene. The yield of the thereby prepared white solid is 64% (0.41 g). ^1^H NMR (400 MHz, CDCl_3_): *δ* = 2.10 (s, 3H, C[7a]–H_3_), 3.80 (s, 3H, C[8b]–H_3_), 4.42 (s, 1H, N[1]–H), 4.68 (d, *J* = 5.2 Hz, 2H, C[2]–H_2_), 6.56 (dd, *J* = 7.0, 5.2 Hz, 1H, C[2b]–H), 6.83 (dd, *J* = 8.2, 2.1 Hz, 1H, C[4b]–H), 7.03–6.92 (m, 2H, C[5a; 6b]), 7.33–7.19 (m, 2H, C[4a; 5b]), 8.05 (d, *J* = 6.0 Hz, 1H, C[6a]) ppm; ^13^C NMR (101 MHz, CDCl_3_): *δ* = 17.0 (q, C[7a]), 45.9 (q, C[8b]), 55.2 (d, C[2]), 112.6 (d, C[2b]), 113.0 (d, C[4b]), 113.5 (d, C[5a]), 116.7 (s, C[3a]), 120.1 (d, C[6b]), 129.6 (d, C[5b]), 137.0 (d, C[4a]), 141.6 (s, C[1b]), 145.25 (d, C[6a]), 156.6 (s, C[3b]), 159.86 (s, C[3a]) ppm.

#### 3-Methyl-*N*-[4-(trifluoromethyl)benzyl]pyridin-2-amine (**17**)

[20] The reaction was carried out according to general procedure C with 67 mg Pd(OAc)_2_ (0.3 mmol, 0.02 equiv.), 187 mg rac. BINAP (0.3 mmol, 0.02 equiv.), 7.26 g K_2_CO_3_ (52.5 mmol, 3.5 equiv.), 1.91 g 2-chloro-3-methylpyridine (15 mmol, 1 equiv.), 3.15 g 4-(trifluoromethyl)benzylamine (18 mmol, 1.2 equiv.), and 38 cm^3^ toluene. The yield of the thereby prepared yellow crystals is 81% (3.24 g).

#### Ethyl 5-[(3-methylpyridin-2-yl)amino]-5-phenylpentanoate (18, C_19_H_24_N_2_O_2_)

The reaction was carried out according to general procedure A with 100 mg **1** (0.50 mmol, 1 eq.), 293 mg ethyl 4-bromobutyrate (1.50 mmol, 3 eq.), 311 mg K_2_CO_3_ (2.25 mmol, 4.5 eq.), and 12 mg [RhCl(cod)]_2_ (0.025 mmol, 0.05 eq.) in 2 cm^3^ dry and degassed toluene. The reaction mixture was heated for 22 h at 160 °C. The general work-up procedure B for C–H activation reactions was followed. The combined organic layers were evaporated and the resulting crude product was purified by flash column chromatography (LP/EtOAc, 9 g SiO_2_, flowrate 15 cm^3^/min) using a gradient which varies the solvents from 0% to 5% EtOAc within 45 min. Drying under reduced pressure delivered **18** in 26% (41 mg) yield as dark yellowish oil. TLC: *R*_f_ = 0.16 (LP/EtOAc 10:1); ^1^H NMR (400 MHz, CDCl_3_): *δ* = 1.05–1.25 (m, 3H, C[9]–H_3_), 1.46–1.95 (m, 4H, C[3, 4]–H_2_), 2.02 (s, 3H, C[7a]–H_3_), 2.11–2.35 (m, 2H, C[5]–H_2_), 4.01 (q, *J* = 7.1 Hz, 2H, C[8]–H_2_), 4.38 (d, *J* = 7.7 Hz, 1H, N–H), 5.19 (q, *J* = 7.1 Hz, 1H, C[2]–H), 6.38 (dd, *J* = 7.1, 5.1 Hz, 1H, C[5a]–H), 7.07–7.15 (m, 2H, C[4a; 4b]–H), 7.17–7.37 (m, 4H, C[2b; 3b; 5b; 6b]–H), 7.85 (dd, *J* = 5.2, 1.7 Hz, 1H, C[6a]–H) ppm; ^13^C NMR (101 MHz, CDCl_3_): *δ* = 14.3 (q, C[9]), 17.1 (q, C[7a]), 21.8 (t, C[3]), 34.0 (t, C[4]), 36.7 (t, C[5]), 54.3 (d, C[2]), 60.3 (d, C[8]), 112.7 (d, C[5a]), 116.4 (s, C[3a]), 126.5 (d, C[2b; 6b]), 126.9 (d, C[4b]), 128.5 (d, C[3b; 5b]), 136.9 (d, C[4a]), 144.0 (s, C[1b]), 145.4 (d, C[6a]), 156.1 (s, C[2a]), 173.5 (s, C[6]) ppm; GC–MS: retention time: 8.10 min; main fragments: *m/z* (%) = 312 (M^+^, 16), 267 (10), 211 (42), 197 (100), 117 (10), 108 (25), 91 (15), 65 (16).

#### Ethyl 6-[(3-methylpyridin-2-yl)amino]-6-phenylhexanoate (19, C_20_H_26_N_2_O_2_)

The reaction was carried out according to general procedure A with 100 mg **1** (0.50 mmol, 1 eq.), 314 mg ethyl 5-bromopentanoate (1.50 mmol, 3 eq.), 311 mg K_2_CO_3_ (2.25 mmol, 4.5 eq.), and 12 mg [RhCl(cod)]_2_ (0.025 mmol, 0.05 eq.) in 2 cm^3^ dry and degassed toluene. The reaction mixture was heated for 22 h at 160 °C. The general work-up procedure B for C–H activation reactions was followed using a gradient which varies the solvents from 0% to 10% EtOAc within 45 min. Drying under reduced pressure delivered **19** in 35% (56 mg) yield. TLC: *R*_f_ = 0.16 (LP/EtOAc 10:1); ^1^H NMR (400 MHz, CDCl_3_): *δ* = 1.13 (t, *J* = 7.1 Hz, 3H, C[10]–H_3_), 1.17–1.47 (m, 2H, C[4]–H_2_), 1.58 (p, *J* = 7.6 Hz, 2H, C[5]–H_2_), 1.70–1.95 (m, 2H, C[3]–H_2_), 2.02 (s, 3H, C[7a]–H_3_), 2.19 (t, *J* = 7.5 Hz, 2H, C[6]–H_2_), 4.09 (q, *J* = 7.1 Hz, 2H, C[9]–H_2_), 4.39 (d, *J* = 7.8 Hz, 1H, N–H), 5.26 (q, *J* = 7.3 Hz, 1H, C[2]–H), 6.47 (dd, *J* = 7.1, 5.1 Hz, 1H, C[5a]–H), 7.15–7.24 (m, 2H, C[4a; 4b]–H), 7.27–7.44 (m, 4H, C[2b; 3b; 5b; 6b]–H), 7.95 (dd, *J* = 5.2, 1.8 Hz, 1H, C[6a]–H) ppm; ^13^C NMR (101 MHz, CDCl_3_): *δ* = 14.2 (q, C[10]), 17.1 (q, C[7a]), 24.9 (t, C[5]), 25. 9 (t, C[4]), 34.2 (t, C[3]), 37.0 (t, C[6]), 54.6 (d, C[2]), 60.2 (t, C[9]), 112.6 (d, C[5a]), 116.4 (s, C[3a]), 126.5 (d, C[2b; 6b]), 126. 9 (d, C[4b]), 128. 5 (d, C[3b; 5b]), 137.0 (d, C[4a]), 144.1 (d, C[6a]), 155.9 (s, C[2a]), 173. 7 (s, C[7]) ppm; GC–MS: retention time: 8.41 min; main fragments: *m/z* (%) = 326 (M^+^, 12), 281 (9), 197 (100), 108 (20), 91 (19), 65 (10).

#### *N*-[1-(3-Methoxyphenyl)pentyl]-3-methylpyridin-2-amine (20, C_18_H_24_N_2_O)

The reaction was carried out according to general procedure A with 114 mg **16** (0.50 mmol, 1 eq.), 206 mg 1-bromobutane (1.50 mmol, 3 eq.), 311 mg K_2_CO_3_ (2.25 mmol, 4.5 eq.), and 12 mg [RhCl(cod)]_2_ (0.025 mmol, 0.05 eq.) in 2 cm^3^ dry and degassed toluene. The reaction mixture was heated for 22 h at 160 °C. The general work-up procedure B for C–H activation reactions was followed using a gradient which varies the solvents from 0% to 10% EtOAc within 45 min. Drying under reduced pressure delivered **20** in 56% (80 mg) yield. TLC: *R*_f_ = 0.42 (LP/EtOAc 10:1); ^1^H NMR (400 MHz, CDCl_3_): *δ* = 0.90 (t, *J* = 7.0 Hz, 3H, C[6]–H_3_), 1.24–1.52 (m, 4H, C[3, 5]–H_2_), 1.89 (tdt, *J* = 16.4, 9.6, 4.5 Hz, 2H, C[4]–H_2_), 1.89 (tdt, *J* = 16.4, 9.6, 4.5 Hz, 2H, C[19]–H_2_), 2.14 (s, 3H, C[7a]–H_3_), 3.82 (s, 3H, C[8b]–H_3_), 4.42 (s, 1H, N–H), 5.26 (q, *J* = 7.5 Hz, 1H, C[2]–H), 6.50 (dd, *J* = 7.1, 5.1 Hz, 1H, C[4b]–H), 6.78 (ddd, *J* = 8.1, 2.6, 1.1 Hz, 1H, C[5a]–H), 6.93–7.03 (m, 2H, C[2b; 6b]–H), 7.18–7.30 (m, 2H, C[4a; 4b]–H), 7.98 (dd, *J* = 5.1, 1.8 Hz, 1H, C[6a]–H) ppm; ^13^C NMR (101 MHz, CDCl_3_): *δ* = 14.0 (q, C[6]), 17.1 (q, C[7a]), 22.7 (t, C[4]), 28.5 (t, C[3]), 54.7 (d, C[2]), 55.2 (q, C[8b]), 111.8 (d, C[2b]), 112.6 (d, C[4b]), 112.7 (d, C[5b]), 116. 5 (s, C[3a]), 118.9 (d, C[6b]), 129.4 (d, C[5a]), 137.1 (d, C[4a]), 145.0 (d, C[6a]), 146.2 (s, C[1b]), 155.9 (s, C[3b]), 159. 7 (s, C[2a]) ppm; GC–MS: retention time: 8.41 min; main fragments: *m/z* (%) = 326 (M^+^, 12), 281 (9), 197 (100), 108 (20), 91 (19), 65 (10).

#### *N*-[1-(3-Methoxyphenyl)undecyl]-3-methylpyridin-2-amine (21, C_24_H_36_N_2_O)

The reaction was carried out according to general procedure A with 114 mg **16** (0.50 mmol, 1 eq.), 332 mg 1-bromodecane (1.50 mmol, 3 eq.), 311 mg K_2_CO_3_ (2.25 mmol, 4.5 eq.), and 12 mg [RhCl(cod)]_2_ (0.025 mmol, 0.05 eq.) in 2 cm^3^ dry and degassed toluene. The reaction mixture was heated for 22 h at 160 °C. The general work-up procedure B for C–H activation reactions was followed using a gradient which varies the solvents from 0% to 5% EtOAc within 45 min. Drying under reduced pressure delivered **21** in 56% (102 mg) yield. TLC: *R*_f_ = 0.65 (LP/EtOAc 10:1); ^1^H NMR (400 MHz, CDCl_3_): *δ* = 0.91 (t, *J* = 6.9 Hz, 3H, C[12]–H_3_), 1.19–1.51 (m, 16H, C[4–11]–H_2_), 1.81–1.97 (m, 2H, C[3]–H_2_), 2.15 (s, 3H, C[7a]–H_3_), 3.82 (s, 3H, C[8b]–H_3_), 4.41–4.48 (m, 1H, N–H), 5.27 (q, *J* = 7.3 Hz, 1H, C[2]–H), 6.51 (dd, *J* = 7.1, 5.1 Hz, 1H, C[5a]–H), 6.75–6.83 (m, 1H, C[4b]–H), 6.94–7.04 (m, 2H, C[5b; 6b]–H), 7.25 (q, *J* = 7.5 Hz, 2H, C[2b; 4a]–H), 7.99 (dd, *J* = 5.2, 1.8 Hz, 1H, C[6a]–H) ppm; ^13^C NMR (101 MHz, CDCl_3_): *δ* = 14.1 (q, C[12]), 17.1 (q, C[7a]), 22.7 (t, C[11]), 26.4 (t, C[4]), 29.31 (t, C[5]), 29.60 (t, C[6–9]), 31.9 (t, C[10]), 37.5 (t, C[3]), 54.7 (d, C[2]), 55.2 (q, C[8b]), 111. 8 (d, C[6b]), 112.5 (d, C[4b]), 112.6 (d, C[5a]), 116.4 (s, C[3a]), 118.8 (d, C[2b]), 129. 4 (d, C[5a]), 137.6 (d, C[4a]), 145.3 (s, C[1b]), 146.2 (d, C[6a]), 156.6 (s, C[5b]), 159.7 (s, C[2a]) ppm; GC–MS: retention time: 9.48 min; main fragments: *m/z* (%) = 368 (M^+^, 9), 241 (21), 227 (100), 207 (6), 108 (26), 91 (6) 65 (5).

#### 3-Methyl-*N*-[1-(*o*-tolyl)pentyl]pyridin-2-amine (22, C_18_H_24_N_2_)

The reaction was carried out according to general procedure A with 100 mg **15** (0.47 mmol, 1 eq.), 206 mg 1-bromobutane (1.50 mmol, 3.2 eq.), 311 mg K_2_CO_3_ (2.25 mmol, 4.5 eq.), and 12 mg [RhCl(cod)]_2_ (0.025 mmol, 0.05 eq.) in 2 cm^3^ dry and degassed toluene. The reaction mixture was heated for 22 h at 160 °C. The general work-up procedure B for C–H activation reactions was followed using a gradient which varies the solvents from 0% to 5% EtOAc within 1 h. Thereafter, a gradient that varies the solvents from 5% to 10% EtOAc was applied. Drying delivered **22** in 47% (60 mg) yield as colorless oil. TLC: *R*_f_ = 0.6 (LP/EtOAc 10:1); ^1^H NMR (400 MHz, CDCl_3_): *δ* = 0.87 (t, *J* = 7.0 Hz, 3H, C[6]–H_3_), 1.19–1.46 (m, 4H, C[4, 5]–H_2_), 1.74–1.99 (m, 2H, C[3]–H_2_), 2.11 (s, 3H, C[7a]–H_3_), 2.32 (s, 3H, C[7b]–H_3_), 4.39 (s, 1H, (m, 1H, N–H)), 5.22 (q, *J* = 7.5 Hz, 1H, C[2]–H), 6.47 (dd, *J* = 7.1, 5.1 Hz, 1H, C[5a]–H), 7.12 (d, *J* = 7.8 Hz, 2H, C[4a;4b]), 7.23–7.16 (m, 1H, C[2b]), 7.27 (d, *J* = 8.1 Hz, 2H, C[3b; 5b]), 7.96 (dd, *J* = 5.1, 1.8 Hz, 1H, C[6a]–H) ppm; ^13^C NMR (101 MHz, CDCl_3_): *δ* = 14.1 (q, C[12]), 17.2 (q, C[7a]), 21.2 (t, C[7b], 22.8 (t, C[4]), 28.7 (t, C[5]), 37.3 (t, C[3]), 54.5 (d, C[2]), 112.5 (d, C[5a]), 116.4 (s, C[3a]), 126.6 (d, C[2b]), 129.2 (d, C[4b]), 136.6 (d, C[4a]), 137.0 (d, C[3b]), 141.6 (s, C[1b]), 156.2 (d, C[5b]) ppm; GC–MS: retention time: 8.88 min; main fragments: *m/z* (%) = 352 (M^+^, 5), 225 (8), 211 (100), 108 (19), 92 (22) 65 (4).

#### 3-Methyl-*N*-[1-(*o*-tolyl)undecyl]pyridin-2-amine (23, C_24_H_36_N_2_)

The reaction was carried out according to general procedure A with 106 mg **17** (0.50 mmol, 1 eq.), 332 mg 1-bromodecane (1.50 mmol, 3 eq.), 311 mg K_2_CO_3_ (2.25 mmol, 4.5 eq.), and 12 mg [RhCl(cod)]_2_ (0.025 mmol, 0.05 eq.) in 2 cm^3^ dry and degassed toluene. The reaction mixture was heated for 22 h at 160 °C. The general work-up procedure B for C–H activation reactions was followed using a gradient which varies the solvents from 0% to 5% EtOAc within 45 min. Drying under reduced pressure delivered **23** in 25% (44 mg) yield. TLC: *R*_f_ = 0.8 (LP/EtOAc 5:1); ^1^H NMR (400 MHz, CDCl_3_): *δ* = 0.92–0.86 (m, 3H, C[12]–H_3_), 1.39–1.20 (m, 16H, C[4–11]–H_2_), 1.97–1.75 (m, 2H, C[3]–H_2_), 2.10 (s, 3H, C[7a]–H_3_), 2.50 (s, 3H, C[7b]–H_3_), 4.53–4.30 (m, 1H, N–H), 5.49 (q, *J* = 6.4 Hz, 1H, C[2]–H), 6.47 (dd, *J* = 7.1, 5.1 Hz, 1H, C[5a]–H), 7.23–7.08 (m, 4H, C[2b–5b]–H), 7.36–7.30 (m, 1H, C[4a]–H), 7.97 (dd, *J* = 5.2, 1.8 Hz, 1H, C[6a]–H) ppm; ^13^C NMR (101 MHz, CDCl_3_): *δ* = 14.2 (q, C[12]), 17.1 (q, C[7a]), 19.6 (q, C[7b]), 22.7 (t, C[11]), 26.5 (t, C[9]), 29.47 (t, C[8]), 29.56 (t, C[7]), 29.63 (t, C[5]), 29.73 (t, C[6]), 31.9 (t, C[4]), 34.5 (t, C[10]), 36.8 (t, C[3]), 51.0 (d, C[2]), 112.4 (s, C[6b]), 116.1 (s, C[3a]), 124. 8 (d, C[5a]), 126.1 (d, C[4b]), 126.5 (d, C[2b]), 130.5 (d, C[3b]), 136.2 (d, C[4a]), 142.8 (s, C[1b]), 145.4 (d, C[6a]), 156.0 (d, C[5b]), 174.0 (s, C[2a]) ppm; GC–MS: retention time: 8.88 min; main fragments: *m/z* (%) = 352 (M^+^, 5), 225 (8), 211 (100), 108 (19), 92 (22) 65 (4).

#### 3-Methyl-*N*-[1-[4-(trifluoromethyl)phenyl]pentyl]pyridin-2-amine (24, C_18_H_21_F_3_N_2_)

The reaction was carried out according to general procedure A with 133 mg **17** (0.50 mmol, 1 eq.), 206 mg 1-bromobutane (1.50 mmol, 3 eq.), 311 mg K_2_CO_3_ (2.25 mmol, 4.5 eq.), and 12 mg [RhCl(cod)]_2_ (0.025 mmol, 0.05 eq.) in 2 cm^3^ dry and degassed toluene. The reaction mixture was heated for 22 h at 160 °C. The general work-up procedure B for C–H activation reactions was followed using a gradient which varies the solvents from 0% to 5% EtOAc within 45 min. Drying under reduced pressure delivered **24** in 50% (81 mg) yield as pale yellowish oil. TLC: *R*_f_ = 0.41 (LP/EtOAc 10:1); ^1^H NMR (400 MHz, CDCl_3_): *δ* = 0.89 (t, *J* = 6.9 Hz, 3H, C[6]–H_3_), 1.18–1.49 (m, 4H, C[4, 5]–H_2_), 1.77–1.96 (m, 2H, C[3]–H_2_), 2.16 (s, 3H, C[7a]–H_3_), 4.48 (s, 1H, N–H), 5.31 (p, *J* = 7.4 Hz, 1H, C[2]–H), 6.50 (dd, *J* = 7.1, 5.1 Hz, 1H, C[5a]–H), 7.23 (d, *J* = 7.1 Hz, 1H, C[4a]–H), 7.49 (d, *J* = 8.2 Hz, 2H, C[2b; 6b]–H), 7.55 (d, *J* = 8.1 Hz, 2H, C[3b; 5b]–H), 7.91 (dd, *J* = 5.1, 1.8 Hz, 1H, C[6a]–H) ppm; ^13^C NMR (101 MHz, CDCl_3_): *δ* = 14.1 (q, C[6]), 17.2 (q, C[7a]), 22.7 (t, C[5]), 28.6 (t, C[4]), 37.5 (t, C[3]), 54.7 (d, C[2]), 113.1 (d, C[5a]), 116.6 (s, C[3a]), 123.1 (s, C[7b]), 125.5, (d, C[3b; 5b]), 126.9 (d, C[2b; 6b]), 129.3 (s, C[6b]), 137.4 (d, C[4a]), 147.3 (s, C[1b]), 148.8 (d, C[6a]), 155.5 (s, C[2a]) ppm; GC–MS: retention time: 6.94 min; main fragments: *m/z* (%) = 322 (M^+^, 18), 265 (100), 159 (11), 108 (41), 92 (46) 65 (24).

#### 3-Methyl-*N*-[1-[4-(trifluoromethyl)phenyl]undecyl]pyridin-2-amine (25, C_24_H_33_F_3_N_2_)

The reaction was carried out according to general procedure A with 133 mg **17** (0.50 mmol, 1 eq.), 332 mg 1-bromodecane (1.50 mmol, 3 eq.), 311 mg K_2_CO_3_ (2.25 mmol, 4.5 eq.), and 12 mg [RhCl(cod)]_2_ (0.025 mmol, 0.05 eq.) in 2 cm^3^ dry and degassed toluene. The reaction mixture was heated for 22 h at 160 °C. The general work-up procedure B for C–H activation reactions was followed using a gradient which varies the solvents from 0% to 5% EtOAc within 45 min. Drying under reduced pressure delivered **25** in 62% (126 mg) yield. TLC: *R*_f_ = 0.79 (LP/EtOAc 10:1); ^1^H NMR (400 MHz, CDCl_3_): *δ* = 0.87 (t, *J* = 6.8 Hz, 3H, C[12]–H_3_), 1.19–1.39 (m, 16H, C[7–11]–H_2_), 1.78–1.94 (m, 2H, C[3]–H_2_), 2.16 (s, 3H, C[7a]–H_3_), 4.47 (d, *J* = 7.0 Hz, 1H, N–H), 5.30 (q, *J* = 7.2 Hz, 1H, C[2]–H), 6.50 (dd, *J* = 7.1, 5.2 Hz, 1H, C[6a]–H), 7.23 (d, *J* = 7.1 Hz, 1H, C[4a]–H), 7.48 (d, *J* = 8.1 Hz, 2H, C[2b; 6b]–H), 7.55 (d, *J* = 8.1 Hz, 2H, C[3b; 5b]–H), 7.91 (dd, *J* = 5.1, 1.7 Hz, 1H, C[6a]–H) ppm; ^13^C NMR (101 MHz, CDCl_3_): *δ* = 14.1 (q, C[12]), 17.0 (q, C[7a]), 22.7 (t, C[11]), 26.3 (t, C[10]), 29.3 (t, C[8]), 29.5 (t, C[6, 7]), 29.6 (t, C[5, 9]), 31.9 (t, C[4]), 37.6 (t, C[3]), 54.6 (d, C[2]), 113.0 (d, C[5a]), 122.9 (s, C[7b]), 125.3 (d, C[3b; 5b]), 126.8 (d, C[2b; 6b]), 128.7 (s, C[3a]), 129.1 (s, C[4b]), 137.8 (d, C[4a]), 145.0, (s, C[1b]), 148.8 (d, C[6a]), 155.5 (s, C[2a]) ppm; GC–MS: retention time: 8.52 min; main fragments: *m/z* (%) = 406 (M^+^, 11), 279 (34), 265 (100), 159 (8), 108 (64), 92 (30) 65 (11).
